# Cold Anaphylaxis in Children: Italian Case Series and Review of the Literature

**DOI:** 10.3390/diseases11040143

**Published:** 2023-10-18

**Authors:** Leonardo Tomei, Francesca Saretta, Stefania Arasi, Lucrezia Sarti, Amelia Licari, Mattia Giovannini, Simona Barni, Giulia Liccioli, Valeria Tallarico, Alessandra Piccorossi, Carlo Caffarelli, Elio Novembre, Francesca Mori

**Affiliations:** 1 Allergy Unit, Meyer Children’s Hospital IRCCS, 50139 Florence, Italy; 2 Department of Health Sciences, University of Florence, 50139 Florence, Italy; 3 Pediatric Department, Latisana-Palmanova Hospital, Azienda Sanitaria Universitaria Friuli Centrale, 33100 Udine, Italy; 4 Translational Research in Pediatric Specialties Area, Division of Allergy, Bambino Gesù Children’s Hospital IRCCS, 00165 Rome, Italy; 5 Pediatric Clinic, Fondazione IRCCS Policlinico San Matteo, University of Pavia, 27100 Pavia, Italy; 6 Pediatric Unit, University Hospital Renato Dulbecco, 88100 Catanzaro, Italy; 7 Pediatric Unit, Bufalini Hospital, AUSL Romagna, 47521 Cesena, Italy; 8 Pediatric Clinic, Medicine and Surgery Department, Azienda Ospedaliero-Universitaria, University of Parma, 43126 Parma, Italy

**Keywords:** pediatrics, chronic urticaria, cold urticaria, anaphylaxis

## Abstract

Chronic urticaria (CU) is one of the most common skin disorders worldwide. Among the inducible subgroup of CU, cold urticaria (ColdU) can affect both children and adults and is the only type associated with the risk of anaphylaxis without cofactors. In the scientific literature, data about cold anaphylaxis (ColdA) are poor, especially at pediatric age, and little is known about risk factors associated with the onset of systemic reactions and about the criteria for prescribing adrenaline auto-injectors (AAIs) in these patients. We describe the clinical characteristics and management of a case series of 21 patients with a history of ColdA, and we compare them with the pediatric case reports and case series published so far. On the basis of the scientific literature and of our case series of patients, we suggest that AAI should be prescribed to all high-risk patients: those with urticaria caused by cold-water immersion, oropharyngeal reactions, and with a previous history of systemic symptoms or anaphylaxis.

## 1. Introduction

### 1.1. Definition

**Figure 1:**
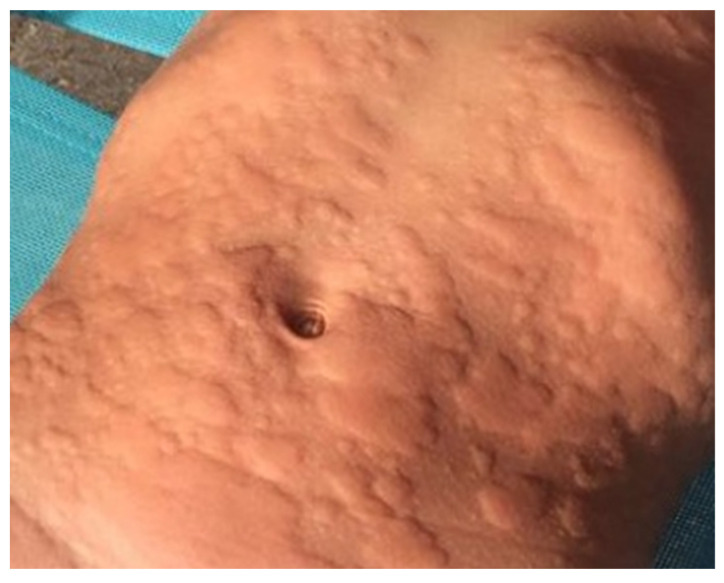
ColdU in a 5-year-old boy.

Urticaria is one of the most common skin disorders worldwide, with 20% of the population affected at least once in a lifetime. It appears more frequently as acute urticaria (AU) in children and as chronic urticaria (CU) in adults [1]. Cold urticaria (ColdU) was first described by Frank in 1792 [2] and belongs to the inducible subgroup of CU (CIndU) [3]; patients with ColdU usually present recurrent episodes with intercurrent periods without clinical manifestations (Figure 1). The episodes are typically triggered by exposure to cold objects, ranging from liquids such as water, solids such as food or ice, and even air. In children, ColdU is estimated to account for 20–30% of all CIndU [4] and is the only type associated with the risk of anaphylaxis without the occurrence of cofactors [5].

The severity of ColdU was defined by Wanderer et al. [6,7]. According to the classification, the clinical patterns of ColdU reflecting the signs and symptoms severity were defined as follows: (a) localized urticaria and/or angioedema (grade 1), (b) systemic reactions characterized by one or more episodes of generalized urticaria or angioedema without hypotensive clinical manifestations (grade 2), and (c) severe systemic reactions with one or more episodes of generalized urticaria or angioedema associated with hypotension, dizziness, fainting, disorientation, or shock (grade 3).

Cold anaphylaxis (ColdA) is defined as an acute cold-induced involvement of the skin and/or visible mucosal tissue, and at least one of the following: cardiovascular manifestations, difficulty breathing, or gastrointestinal symptoms [8,9]. The risk of anaphylaxis in ColdU and the individuation of risk factors for ColdA need to be studied, especially in children, who show a longer duration, a worse prognosis, and a higher risk of ColdA [9]. Some pediatric case series on ColdA have been published, but no common risk factors have been clearly identified.

Several hypotheses have been published regarding the etiopathogenesis of ColdA. In a multi-center study including 551 adult and pediatric patients, the cold stimulation test (CST) was positive in 75% and among these patients, 37% had an episode of ColdA [9]. In tropical climates, the more common trigger for ColdA is the exposure to cold air, while in colder climates exposure to cold foods, liquids, and surfaces is the trigger, suggesting that continued exposure to cold air could determine desensitization to that stimulus, which was proposed as a possible treatment before [10,11].

Additionally, the authors identified five risk factors for ColdA in typical ColdU: a previous systemic reaction to a Hymenoptera sting (*p* = 0.017), presence of angioedema (*p* < 0.001), oropharyngeal/laryngeal clinical manifestations (*p* = 0.001), itchy earlobes (*p* = 0.005), and asthma comorbidity (*p* = 0.015). No specific risk factors were identified for the pediatric subgroup.

Moreover, the question of when to prescribe an adrenaline auto-injector (AAI) to patients with ColdU has not been answered yet. The above-mentioned multi-center study [12] furtherly underlines the need for a specific management of patients with ColdU related to the risk of anaphylaxis compared to other subtypes of CIndU and CSU. In the multi-center study, AAI was prescribed only in 37% of adults and 30% of children, and of those, only 20% of children and 8% of adults have received an AAI. A different approach has been taken in a Canadian Allergy Practice case series [13], where AAI was prescribed to 32% of ColdU patients, regardless of previous episodes of ColdA (only 2 among 50 studied patients). However, that paper does not provide additional information on possible risk factors for ColdA or indications for prescribing AAI in ColdU, which remain elusive.

### 1.2. Prevalence

In a recent systematic review and meta-analysis that comprises both adult and pediatric patients, Prosty et al. [14] assessed the pooled prevalence of ColdU among CU and CIndU cases, determining them to be 8% and 26%, respectively. Of nineteen examined articles, five enrolled only adults, five only children, and nine both populations. They have also estimated the point prevalence of ColdU to be 0.056%, or approximately 6/10,000 people worldwide at any given time. Although ColdU is rare, it is a potentially life-threatening condition. Among all analyzed studies including adults and children, eleven reported data on anaphylaxis, with a prevalence ranging between 0% and 34.3% and no recorded fatalities.

ColdA epidemiology is not well defined, particularly in children, but several case reports and a few case series of ColdA have been published so far, as shown in Table 1 [15,16,17,18,19,20,21,22,23,24].

### 1.3. Pathogenesis

The pathogenesis of ColdU is not well defined yet and many unclear aspects need to be clarified, as accurately described by Maltseva et al. [8]. A hypothesis suggests the presence of IgE autoantibodies that react only at low temperatures against specific skin antigens, but none has been identified yet. The role of mediators released during ColdU attacks has been under investigation since the 1980s [18] and has been reviewed very recently by Kulthanan et al. [25]. The authors conclude that histamine has a “key pathogenic role”, but more studies are needed to identify other potential factors in the pathogenesis of CIndU. Meyer et al. [26] have characterized the changes in microcirculation after mast cell degranulation in ColdU. The authors have demonstrated that, in primary ColdU, mast cells are activated by cold triggers with subsequent degranulation and extensive histamine release. This suggests that mast cells and histamine release are the principal effectors in ColdU, inducing, e.g., increased blood flow, vasodilation, and collateral circulation recruitment. Also, these observed modifications show an apparent response to antihistamine administration. Other studies have focused on the role of cold agglutinins and cryoglobulins, but results are discordant even among publications of the same group, and most importantly, none of them have specifically analyzed pediatric populations. In a single-center study [27], patients with ColdU were tested for cryoproteins: 16/35 (46%) had a positive cold agglutinin test and 9/33 (27%) had a positive cryoglobulin test. Conversely, in a meta-analysis by Ginter et al. [28], it appears that only a few ColdU patients have cryoproteins. Therefore, further studies are necessary to highlight the role of these proteins in the pathogenesis of the disease.

As mentioned above, mast cells have a key role in ColdU (as well as in all types of urticaria), since their activation and degranulation being the main cause of wheals. In ColdU, the low temperature provokes the degranulation of mast cells [29]. Already in the 1970s, different authors found that following mast cell degranulation there was an increase in several mediators, such as histamine, tumor necrosis factor-α, prostaglandin D2, and platelet-activating factor-like lipid [30,31,32,33]. Different activating receptors (such as FcεRI, MRGPRX2, C5aR, PAR1, PAR2, and CRTh2) and inhibiting receptors (such as Siglec 8, CD200R, CD300a, and FcγRIIb) are expressed on mast cells, and their interactions lead to several cascading pathways as extensively described by Kolkhir P et al. [34]. Moreover, the release of tryptase and cytokines (IL-4, IL-5, IL-13, IL-17, and IL-31) further involves other cells such as eosinophils, basophils, and T cells. 

However, according to Meyer et al. [26], mast cells and histamine are the main cause of wheals while other cells and mediators play only a minor role [35].

The identification of specific cold-induced skin antigens remains still unfulfilled. Currently, there is no direct proof of the involvement of IgE antibodies, although some studies have already tried to obtain some indirect evidence. Houser et al. [36] have studied the role of IgE antibodies through passive transfer, which has been demonstrated to be a possible pathogenic cause of ColdU [30]. However, the exact mechanism is still unclear even though omalizumab has been shown to be effective in ColdU [37].

### 1.4. Genetics

In a case series by Ombrello et al. [38], three families with a dominant form of ColdU were studied. Using linkage studies and targeted sequencing, an in-frame deletion in the regulatory domain of this phospholipase Cγ2 (PLCG2) was individuated as a possible genetic cause. PLCG2 encodes an enzyme involved in the immunological cascade and it is present in mast cells, B cells, and natural killer (NK) cells. In these patients, at low temperatures, mast cells degranulated, and B cells and NK cells increased their activation, while at 37 °C no degranulation was observed, and B cells and NK cells had a reduced activity.

### 1.5. Diagnosis

#### 1.5.1. Physical Tests

Physical stimuli such as the ice cube test, TempTest©, or immersion test can be used as CST to confirm the diagnosis of ColdU [39]. According to the EAACI/GA^2^LEN/EDF/UNEV consensus recommendations on diagnostic testing of CIU, the ice cube test consists of placing an ice cube covered in a plastic bag/foil on a patient’s skin (usually the medial forearm surface) for 5 min. If positive, wheals should be observed within 10–20 min. The reported sensitivity is 53–83% with a specificity of 97–100% [40,41]. The TemptTest© has been recently developed and standardized and it exposes the patient’s arm to a range of temperatures (4–44 °C) for 5 min; a U-shaped aluminum stencil indicates the temperature continuously and determines at which temperature the symptoms/signs appear and wheals are triggered. This test could be used in children, too [16]. The reported sensitivity of TempTest© is 93–100%, with a specificity of 100% [40,42]. The immersion test consists of placing the patient’s arm in a cold-water bath, but this should be done with caution because it can elicit anaphylaxis [39]. Two different subtypes of ColdU are reported, according to the response to CST. The typical variant shows that cold-induced wheals appear in the exposed skin immediately or within 5–15 min after cold exposure. This variant could be primary or secondary, e.g., to infections, cryoglobulinemia, drugs, vasculitis, and neoplasms. The atypical variant shows atypical cold-induced wheals which could also appear far from the cold application site, and an atypical response to the CST. Different types of atypical variants are reported, such as systemic atypical ColdU, localized ColdU, localized cold reflex urticaria, delayed ColdU, cold-induced cholinergic urticaria, and cold-dependent dermographism [8].

#### 1.5.2. Differential Diagnosis

It is essential, before formalizing a ColdU diagnosis, to determine if there exists any primary underlying cause. There are, indeed, some heritable forms of ColdU, named CAPS (cryopyrin-associated autoinflammatory syndromes) which are rare autoimmune disorders characterized by an IL-1b overproduction determined by mutations in the gene NLR family pyrin domain containing 3 (NLRP3) [43]. CAPS include the familial cold autoinflammatory (or urticaria) syndrome (FCAS/FCU), Muckle–Wells syndrome, and the neonatal-onset multisystem inflammatory disease (also known as CINCA chronic infantile neurologic cutaneous articular syndrome). Clinically, CAPS are characterized by episodes of fever, urticaria, and/or angioedema that may arise spontaneously or in response to cold triggers, fatigue, and other stressors. Patients with these clinical manifestations should be screened for possible CAPS, especially infants and young children.

#### 1.5.3. Laboratory Tests

There is no test specific for the diagnosis of ColdU, but laboratory tests can be useful to exclude differential diagnosis on the basis of the history and the clinical presentation of the patient. The list of exams proposed by Mari and Banks [44] includes blood tests [complete blood cell count with differential, C-reactive protein, erythrocyte sedimentation rate, circulating cryoglobulins, cold agglutinins, serum C4, serum amyloid A, genetic testing (NLRP3 gene mutation), and skin biopsy. Additionally, in severe cases of ColdU, basal serum tryptase should be added to the diagnostic workout in order to exclude an underlying clonal mast cell disorder [45].

### 1.6. Desensitization

In a study by von Mackensen YA et al. [46], a cold tolerance induction was proposed in a group of 23 people (age range 16–66 years). In two patients, treatment was suspended due to a lack of compliance and massive generalized urticaria and anaphylaxis. The nine patients who responded to the follow-up interview (up to 15 years later) confirmed that the reached tolerance was maintained as long as they continued with the cold bath exposure, but only one patient was able to continue for six months. The rest of the patients stopped due to side effects, such as paresthesia, cold chest, and headaches.

### 1.7. Aims of the Study

This study aims to compare the clinical characteristics and management of our patients with a history of ColdA with the pediatric case reports and case series published so far and to identify some features that could suggest when an AAI prescription is needed.

## 2. Material and Methods

We retrospectively reviewed the electronic charts of patients younger than 18 years old with a history of ColdU from January 2015 to December 2022. ColdA was diagnosed according to the previously mentioned criteria; patients with diagnosis or suspected CAPS were excluded from the study. We collected data concerning demographic characteristics, clinical manifestations, diagnostic workups, and treatment for each patient. Blood tests were performed when clinically deemed appropriate. An absolute blood eosinophil value > 600 × 10^3^ cells/µL, total IgE level > 240 IU/mL, tryptase > 13.5 μg/mL, and CRP > 0.5 mg/dL were categorized as elevated based on the reference level of our laboratory. Additionally, disease progression, response to treatment, and the necessity of using AAI were documented on the basis of clinical records on the electronic charts. ColdU was considered resolved when no symptoms appeared after cold exposure for at least one year without any treatment.

Descriptive statistics were calculated using Microsoft Excel.

Written informed consent was obtained from the parents or legal guardians of patients.

## 3. Results

### 3.1. Demographic Characteristics and Comorbidities

A total of 21 pediatric patients who presented at least one episode of ColdA were included in the study, 12 (57.14%) were male and 9 (42.86%) were female. The median age at the onset of the disease was 9.20 years (IQR 6.25–11.40 years), and two patients had the first episode of ColdA very early in their life, before the age of three. The median age at the time of the first pediatric allergological evaluation was 10.60 years (IQR 7.25–13.25). Less than half of the patients (8, 38.10%), had a first-degree familiar history of atopy, while two patients (9.52%) had a first-degree familiar history of CSU and the other two of autoimmunity diseases.

Regarding the personal clinical history of atopy, about a quarter of patients (5, 23.81%) had allergic asthma, four patients (19.05%) had rhino-conjunctivitis and three patients (14.29%) had atopic dermatitis. Only one patient had food allergies to milk, eggs, and nuts, while no one reported drug or Hymenoptera venom allergy.

One-third of children (7, 33.33%) had other concomitant types of CU: two had CSU, while five had other CIndUs (two heat urticaria, one delayed pressure urticaria, one solar urticaria, one exercise-induced urticaria). Finally, one patient had concomitant juvenile idiopathic arthritis (JIA).

Regarding the first episode of ColdA, only one-third of patients (7, 33.33%) underwent medical evaluation after the episode. One child received adrenaline to treat the ColdA, while the others improved spontaneously or with the administration of antihistamines or corticosteroids.

### 3.2. Reaction Triggers

The vast majority of patients (19, 90.48%) had ColdA after complete cold immersion, while two patients (9.52%) developed the reaction after cold-air exposure. Moreover, most patients (16, 76.19%) developed anaphylaxis after previous episodes of ColdU, while only five patients (23.81%) developed ColdA as a first manifestation. The patients also had a history of grade 1 or grade 2 episodes of cold urticaria either before or after the episode of anaphylaxis; six patients (28.57%) developed ColdU in response to a single trigger, while most children (15, 71.43%) developed clinical manifestations when exposed to multiple triggers. Of patients that reacted to a single trigger, in five children the trigger was cold water, while one patient presented urticaria only after exposure to cold air. Overall, triggers of low-grade reactions were cold water (20 patients, 95.24%), cold air (14 patients, 66.67%), ingestion of cold substances (4 patients, 19.05%), and contact with cold surfaces (3 patients, 14.29%).

### 3.3. Clinical Manifestations of ColdA

Regarding the clinical manifestation of ColdA (Table 2), all patients developed cutaneous signs and symptoms with the onset of generalized urticaria, while angioedema was described in less than a third of patients (6, 28.57%). Following cutaneous involvement, the most common manifestation involved the cardiovascular system, with 18 patients (85.71%) reporting syncope/hypotension. More than a third of children developed gastrointestinal clinical manifestations (8 patients, 38.10%), seven presented isolated vomit and one presented vomit, abdominal pain, and diarrhea. Furthermore, five children (23.81%) had respiratory manifestations: two (9.52%) patients reported dyspnea, two cough, and one bronchospasm.

### 3.4. Provocation Test and Laboratory Studies

Diagnosis of ColdA was achieved by the collection of clinical history and by performing CST. Among all patients, the CST result was negative in three children (14.29%), but they had a highly suggestive clinical history, so these patients were considered atypical ColdU.

Laboratory tests were performed in two-thirds of patients (14, 66.67%), and they included, among others: differential blood count, C-reactive protein, basal tryptase, and total IgE. None of the children had abnormal basal tryptase and only one patient had elevated eosinophil count, while elevated total serum IgE was recorded in four patients (19.05%). Antinuclear antibodies (ANA) research was performed in ten children, and was positive in four patients (19.05%), including the patient with JIA; the maximum titer was 1:320. All these children underwent a pediatric rheumatology evaluation that excluded the possibility of an autoinflammatory syndrome.

### 3.5. Management and Follow-Up

In patients diagnosed with ColdA, avoidance of physical stimuli eliciting the reaction was recommended, along with the prescription of as-needed second-generation H1-antihistamines and oral corticosteroids as pharmacological treatment of the clinical manifestations. In addition, in most patients (19, 90.48%) AAI was prescribed.

The median follow-up period was 19.50 months (IQR: 10.25–24.00 months), considering that 3 out of 21 (14.29%) patients were lost at follow-up. In ten patients (47.62%) clinical manifestations of ColdA disappeared during the follow-up, with a mean duration of disease of 21.2 months (SD: 7.38 months). Among those patients, six were male, four were female, and half were atopic. On the other hand, eight patients (38.10%) reported the persistence of the symptoms at the last follow-up. Two of them were atopic and six were non-atopic, and half were male. In these children, the mean age at the time of the onset of symptoms was slightly lower if compared with that of patients that had resolved the disease (6.63 years vs. 10.1 years, respectively). The mean age at the last follow-up was similar in the two groups of patients: 12.50 years in the patients who reported persistence of symptoms and 13.20 years in the children who had resolved the disease. No statistically significant difference was detected in the two groups of patients in terms of sex (*p* = 0.67), presence of atopic comorbidities (*p* = 0.27), or age at the onset of the disease (*p* = 0.0761).

Additionally, in seven patients (33.33%) with at least monthly episodes of ColdU with deterioration of quality of life, alongside pharmacological treatment of signs and symptoms, one or more cycles of omalizumab were administered. Of these, one (14.28%) patient reported complete resolution of clinical manifestations, while three (42.86%) children reported an improvement of ColdU symptoms. Additionally, one patient described complete resolution throughout the therapeutic cycle, with recurrence during wash-out periods. On the other hand, two (28.57%) patients have not benefited from the therapy, with recurrence of urticaria with cold exposure.

Only one patient reported a further episode of ColdA requiring adrenaline during the follow-up period.

## 4. Discussion

The present paper describes the clinical characteristics and management of 21 Italian children and adolescents with a history of ColdA. In our case series, the most common elicitor of the reaction was water immersion. All the episodes of ColdA were characterized by generalized urticaria, and the most frequent associated clinical manifestation was syncope/hypotension. An AAI was prescribed at first allergological evaluation in all except two cases, but only one patient developed a subsequential episode of ColdA during the follow-up.

Only a few studies have tried to characterize children with ColdU and ColdA (Table S1) [47,48,49,50]. Alangari et al. [49] evaluated 30 children (age < 18 years) over a three-year interval at a pediatric hospital and a private allergy practice. The median age was 12 years, with a median onset age of 7 years. No secondary causes were diagnosed. The ice test was performed on all patients; no differences in clinical presentation or in the response to antihistamines were found between those with positive or negative CST. The authors found that almost half of the children had asthma and allergic rhinitis, and 89.3% had a family history of atopy. A total of 36.7% of children had a systemic reaction, and 72.7% showed neurological clinical manifestations such as dizziness or faintness. In this group, swimming was the common trigger in all but one (cold-air exposure). The authors argued that, given this high percentage of ColdA in ColdU, all patients with ColdU should be warned about this risk and an AAI should be provided.

More recently, Prosty et al. [47] evaluated 52 children with ColdU from 2013 to 2021 and compared data from this group with a pediatric cohort of exclusively chronic spontaneous urticaria (103 children). The median age of signs and symptoms was 9.5 years and 51.9% of the patients were female. The large majority of the patients (90.4%) had typical ColdU. Swimming was the most common trigger (76.3%), followed by cold weather/air (65.8%), and ingestion of cold food or drink (18.4%). About a half of children (48.1%) had other concomitant types of CU; associated atopic diseases were asthma (30.8%), atopic dermatitis (30.8%), allergic rhinitis (21.2%), food allergies (9.6%), and venom allergies (1.9%). The resolution rate was 4.8 per 100 patient-years, lower than CSU. Most children have been treated with second-generation H1-antihistamines with a well-controlled disease course. A total of 17.3% of children showed signs and symptoms suggestive of cold anaphylaxis: shortness of breath in 44.4%, hypotension/syncope in 44.4%, abdominal pain in 22.2%, headache in 22.2%, and fatigue in 11.1%. Only one child required epinephrine treatment, and there were no fatalities or hospitalizations. In ColdA, children’s triggers were swimming in 44.4% and cold air in 44.4%. The authors also found that elevated blood eosinophils (absolute eosinophil number > 0.60 × 10^9^ cells/L) were associated with cold anaphylaxis and suggested that eosinophil counts could be used to screen children at risk for cold anaphylaxis. Finally, Yee et al. [48] analyzed 415 patients (4 months–18.3 years, median age at diagnosis 8.9 years) evaluated for episodes of ColdU. 78.3% of children had atopic diseases and 25.8% had other forms of CU. The male/female ratio was similar, even though a female predominance was observed in older children/adolescents (62.5%). Two-thirds of patients referred localized urticaria, while 14% had diffuse urticaria, and 18.6% experienced anaphylactic reactions (and 7% had more than one episode). The CST was performed in 61.7% of children, and it was positive in 69.9% of them. Interestingly, a positive CST was significantly associated with an increased risk of anaphylaxis. Only 8.9% of children showed disease resolution during the 9 years of study, and all had no previous episodes of anaphylaxis.

We have compared the clinical characteristics with those of children described in only pediatric case reports and case series published so far (Table 1) [15,16,17,18,19,20,21,22,23,24]. In agreement with our results, we can notice that ColdA typically occurs at 8–9 years of age. No vast differences regarding sex prevalence have been reported, while atopic comorbidity seems not to be highly associated with ColdA. Most studies published on children did not specify if they had the first manifestation of ColdU with concurrent anaphylaxis. Few studies reported the recurrence of new grade 3 reactions with a percentage ranging from 4.8 to 37.6%. Alangari et al. [49] reported previous grade 3 reactions as a risk factor for subsequent ColdA. Another study associates the risk for anaphylaxis with the positivity of CST in terms of response to stimulation [6,51] and with the diameter of the wheal [48].

In most pediatric studies, the use of adrenaline is not specified. The severity of ColdA differs from all the other IgE-mediated reactions in children and clinical manifestations affecting the nervous system, with loss of consciousness or dizziness being the most frequently reported with a percentage ranging from 44.4 to 85.7% (Table S1). No fatal ColdA has been described so far.

The most common trigger for anaphylactic reactions was swimming (77.6% to 100%), followed by cold air (8%), contact with cold water, or ingestion of cold food or drink (both 6.3%) (Table S1). Probably, the larger the exposed area and longer the contact with the cold trigger, the higher the risk of grade 3 reactions [26] may be. Consequently, it could be argued that reactions may be due to massive mastocyte degranulation with vasodilation activated by a non-specific mechanism. For that reason, Table 3 proposes some preventive measures, summarizing what is already reported in the pediatric studies published so far on this topic.

As underlined by other authors, the management of all patients evaluated for ColdU requires a careful explanation of all possible risk factors both for ColdU occurrence and especially for ColdA prevention. ColdU urticaria requires constant attention to avoid triggering situations.

Among all types of CIndU, ColdU is the only one associated with life-threatening reactions, especially anaphylaxis, which should be the indication for prescribing an AAI. Therefore, we also agree that precautionary AAI should be prescribed to all high-risk patients: those with urticaria to cold-water immersion, with oropharyngeal reactions, and with a previous history of systemic symptoms or anaphylaxis, as already suggested by Mari and Banks [44].

**Figure 2:**
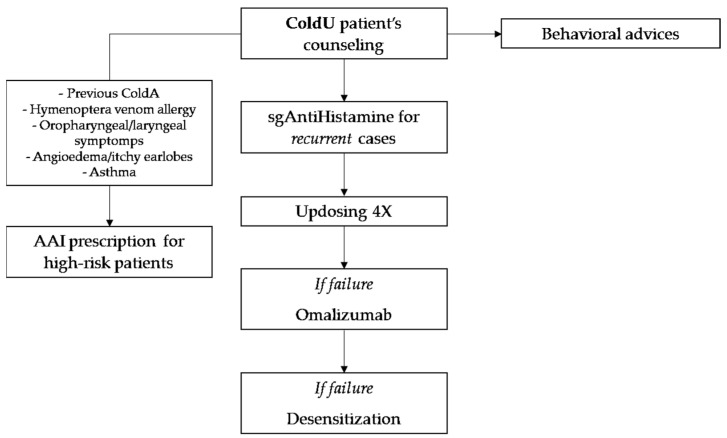
Management for ColdU/ColdA patients from counseling to experimental drugs.

In Figure 2 we provide a possible management pathway for ColdU/ColdA patients. As in CSU [52], omalizumab has proven safe and effective in adult patients with CIndU [53]. Therefore, that drug should also be considered at pediatric age for the treatment of more severe cases of ColdU that do not respond to only antihistamine therapy [54]. In our case series, one or more cycles of omalizumab were administered to seven patients, and all except two of them reported an improvement in ColdU symptoms. Regardless, more studies with a larger number of patients are needed to better evaluate the efficacy of omalizumab for the treatment of ColdU in pediatric age.

ColdU shows a low-resolution rate, with only 10.30% of children in a 5-year follow-up period [32]. The resolution rate seems to be lower in children because, in this age group, there is a higher proportion of atypical ColdU cases [55]. In our population, the ColdU duration was less than 2 years, probably because only two children suffered from atypical ColdU.

## 5. Conclusions

Despite its rarity, ColdU is linked with systemic and potentially life-threatening reactions. From the literature, it is possible to identify only a few preventable risk factors for ColdA. Future studies focusing on phenotyping children at higher risk for anaphylaxis are needed. At the moment, adrenaline should be prescribed to all patients suffering from ColdU to treat grade 3 reactions. New therapeutic options and multicentric works with a vast number of patients included are needed to reach a better knowledge of this disease in order to develop optimal management.

## Figures and Tables

**Table 1:** Case reports and case series of ColdA in children.

Case Description			Management of the Anaphylaxis	AAI Prescription	Ref.
Age, Sex	Trigger	Clinical Manifestation			
11-year-old girl	Swimming in cold water	Urticaria, pallor, loss of consciousness	Oral steroids and antihistamines	Yes	[15]
6-year-old boy	Swimming in cold water	Vomit, pallor, loss of consciousness	Improved spontaneously	Yes	[16]
9-year-old boy	Swimming in cold water	Urticaria, dyspnea, conjunctival hyperemia, blurred vision and loss of strength	IM steroids and IV antihistamine	Yes	[17]
15-year-old girl	Systemic cooling during surgery	Urticaria, angioedema, hypotension, desaturation	IV epinephrine, IV steroids, and antihistamine	No	[18]
9-year-old girl	Walking in seawater	Urticaria, dry throat, dyspnea, loss of consciousness	Improved spontaneously	Yes	[19]
14-year-old girl	Cold drink	Anaphylaxis (unspecified)	Not reported	Not reported	[20]
14-year-old boy	Swimming in cold water				
2-year-old boy	Swimming in cold water				
13-year-old girl	Swimming in cold water	Loss of consciousness, dizziness	Improved spontaneously	Yes	[21]
12-year-old boy	Swimming in cold water	Loss of consciousness, generalized seizure	Improved spontaneously	Yes	[22]
One child with ColdA	Not reported	Not reported	Not reported	Yes	[23]
9-year-old girl	Swimming in cold water	Urticaria, loss of consciousness	Improved spontaneously	Yes	[24]

AAI: adrenaline auto-injector; IM: intramuscular; IV: intravenous.

**Table 2:** Clinical manifestations of ColdU.

	N of patients (%)
Urticaria	21 (100%)
Angioedema	6 (28.57%)
Syncope/hypotension	18 (85.71%)
Gastrointestinal symptoms	8 (38.10%)
	7 vomit
	1 vomit, abdominal pain, diarrhea
Respiratory symptoms	2 (9.52%)
	2 dyspnoea
	2 cough
	1 bronchospasm

**Table 3:** Management recommendations in ColdU and ColdA.

Management Recommendations in ColdU and ColdA
Detailed anamnesis and family counseling
Avoidance of immersion in cold water
Swimming under surveillance
Avoidance of systemic cold exposure (rapid indoor/outdoor passage, refrigerated section at the supermarket, ice skating rink)
Warming of intravenous or irrigation fluids and premedication before surgery
Appropriate selections of clothes in cold weather
Pharmacological therapy (antihistamine and omalizumab)
Desensitization
Epinephrine auto-injector in high-risk cases

## Data Availability

Data available on request due to privacy and ethical restrictions.

## Supplementary Materials

The following supporting information can be downloaded at: https://www.mdpi.com/article/10.3390/diseases11040143/s1, Table S1: Main studies on ColdU including children.

## Abbreviations

AAI: adrenaline auto-injector; ANA: antinuclear antibodies; AU: acute urticaria; CAPS: cryopyrin-associated autoinflammatory syndromes; CIndU: chronic inducible urticaria; ColdA: cold anaphylaxis; ColdU: cold urticaria; CST: cold stimulation test; CU: chronic urticaria; JIA: juvenile idiopathic arthritis.
