# Supplementary material for: Cold Anaphylaxis in Children: Italian Case Series and Review of the Literature
Source: Diseases. 2023 Oct 18;11(4):143. doi: 10.3390/diseases11040143 (PMC10594430; doi:10.3390/diseases11040143)
Supplement: Supplementary file 1 [file diseases-11-00143-s001.zip › diseases-2501571-supplementary.pdf]

Authors	N. of children (N. of children with ColdA, %)	Mean Age	Sex	Atopy					Urticaria		Diagnosis				Triggers			Symptoms					Adrenaline administration
				A	AD	FA	AR	O	CSU/ CINDU	Other Grade 1°-2°-3° reactions	CST (+)	EOS	IgE	AI	Sw	CA	O	S	GI	R	N	O	
Prosty C et al. [47]	**52 (9, 17.3%)**	9.5 yrs*	27F/25M*	16/52 (30.8%) *	16/52 (30.8%) *	5/52 (9.6%) *	11/52 (21.2%) *	1/52 (1.9%) *	CSU 20/52 (42.3%)<br>CINDU 3/52 (5.8%)<br>Both 2/52 (3.8%)<br>*	-	90.4%*	1.38 HR	-	2/52 (3.8%) 1 Celiac disease<br>1 Type I Diabetes *	**4/9 (44.4%)**<br>**Both 1 (11.1%)**	**4/9 (44.4%)**		-	**2/9 (2.2%)**	**4/9 (44.4%)**	**4/9 (44.4%)**<br>**2 headache**<br>**1 fatigue**	-	**1/9 (11.1%)**
Yee CSK et al. [48]	**415 (77, 18.6%)**	8 yrs*	45F/ 32M	194/415 (46.8%) *	102/415 (24.6%) *	139/415 (33.4%) *	247/415 (59.5%) *	59/415 (14.2%) *	CSU 14.2%/ CINDU 18.3% *	**29/77 (37.7%) other Grade 3° reactions**	**84.7%**	45.3% *	68.9% *	15 (3.6%) *	**77.6% (6.3%)WI**	**8%**	**6.3%$ 1.8%$**	-	-	**25%**	**54.5%**	**10.8%#**	**2/14 (14.3%)$**
Alangari AA et al. [49]	**30 (11, 36.7%)**	**8 yrs**	8F/22M	14/30 (46.7%) *	4/30 (13.3%) *	-	15/30 (50%) *	-	CSU 2/30 CINDU 1/30 *	Previous Grade 3 reactions identified as risk factor for anaphylaxis	**6/171 (35.3%)**	-	-	-	**10/11 (90.9%)**	**1/11 (9.1%)**	-	-	-	**5/11 (45.5%)**	**8/11 (72.7%)**	-	-
Azkur D et al. [50]	**15 (1, 6.6%)**	11.2 +-4.5yrs *	10F/5M	1/15 (6.7%) *	0	1/15 (6.7%) *	2/15 (13.3%) *	-	-		12/15 (80%) *	1/15 (6.6%) *	6/15 (40.0%) *	1/15 (6.7%) *	**100%**	-	-	-	-	-	-	-	-
Present study	**21 children with ColdA**	**9.2 yrs**	**9 F/12M**	**5/21 (23.8%)**	**3/21 (14.4%)**	**1/21 (4.8%)**	**4/21 (19.5%)**	-	**CSU 5/ 7/CINDU**	**1/21 subsequent anaphylaxis**	….	**1/21 (4.8%)**	**4/21 (19%)**	**1/21 (4.8%)**	**19/21 (90.5%)**	**2/21 (9.5%)**	-	**21/21 (100%)**	**8/21 (38%)**	**5/21 (23.8%)**	**18/21 (85.7%)**	-	…..

Table S1: main studies on ColdU including children.

*Information regarding all children with Cold Urticaria not specified for Cold Anaphylaxis
yrs= years; A=asthma; AD= atopic dermatitis; FA= food allergy; AR= allergic rhinitis; O= others; CSU= chronic spontaneous urticaria; CINDU= chronic Inducible Urticaria; CST (+)= Cold stimulation test positive; EOS= eosinophils; IgE= Immunoglobulin E; AI= autoimmune diseases; Sw= swimming; CA= cold air; S=skin involvement (urticaria/angioedema); GI= gastrointestinal symptoms; R=respiratory symptoms; N= neurologic symptoms;
# Throat tightness, discomfort
$ Cold Beverages; hospital irrigation with cold fluids; infusion at room temperature Intravenous fluids
WI Without immersion

Azkur not specified symptoms of anaphylaxis
